# The Influence of Haemoglobin A1c Levels on Platelet Aggregation and Platelet Turnover in Patients with Coronary Artery Disease Treated with Aspirin

**DOI:** 10.1371/journal.pone.0132629

**Published:** 2015-07-06

**Authors:** Søs Neergaard-Petersen, Anne-Mette Hvas, Erik Lerkevang Grove, Sanne Bøjet Larsen, Søren Gregersen, Steen Dalby Kristensen

**Affiliations:** 1 Department of Cardiology, Aarhus University Hospital, Aarhus, Denmark; 2 Department of Clinical Biochemistry, Aarhus University Hospital, Aarhus, Denmark; 3 Department of Endocrinology and Internal Medicine MEA, Aarhus University Hospital, Aarhus, Denmark; University of Catanzaro Magna Graecia, ITALY

## Abstract

**Background:**

Hyperglycaemia may attenuate the antiplatelet effect of aspirin and thereby increase the risk of cardiovascular events. We investigated the influence of increased haemoglobin A1c (HbA1c) levels on platelet aggregation and turnover in a large cohort of patients with coronary artery disease (CAD) with type 2 diabetes, prediabetes or no diabetes.

**Methods:**

In this observational study, we included 865 stable CAD patients on 75 mg aspirin as mono-therapy of whom 242 patients had type 2 diabetes and were receiving antidiabetic drugs. Among 623 patients without diabetes, we classified 303 patients with prediabetes (HbA1c ≥5.7–6.4% [39–47 mmol/mol]) naive to antidiabetic drugs. Platelet aggregation was evaluated by the Multiplate Analyzer using arachidonic acid and collagen and by the VerifyNow Aspirin. Platelet turnover was evaluated by immature platelets using flow cytometry and platelet activation by soluble P-selectin.

**Results:**

CAD patients with type 2 diabetes had higher platelet aggregation (all p-values <0.01), platelet turnover (immature platelet count, p<0.01) and platelet activation (p<0.001) than patients without diabetes. CAD patients with prediabetes had increased platelet aggregation (p = 0.02) and platelet count (p = 0.02) compared with patients without diabetes. Increased levels of HbA1c correlated positively with increased platelet aggregation using arachidonic acid (r = 0.19, p<0.0001), collagen (r = 0.10, p<0.01) and VerifyNow (r = 0.15, p<0.0001), and with platelet count (r = 0.08, p = 0.01), immature platelet count (r = 0.11, p<0.001) and soluble P-selectin (r = 0.15, p<0.0001). These associations were mainly evident in non-diabetic and prediabetic CAD patients.

**Conclusions:**

CAD patients with prediabetes and diabetes may have attenuated antiplatelet effect of aspirin compared with CAD patients without diabetes. This may be related to increased platelet count in patients with prediabetes. Increased levels of HbA1c correlated positively, though weakly, with increased platelet aggregation, platelet turnover and platelet activation.

## Introduction

Coronary artery disease (CAD) is a leading cause of morbidity and mortality [1], particularly in type 2 diabetic patients [2]. Importantly, also patients with prediabetes have increased risk of cardiovascular events [3,4]. Low-dose aspirin inhibits platelet aggregation and is a cornerstone in the long-term management of patients with stable CAD [5]. The antiplatelet effect of aspirin can be evaluated by measurements of platelet aggregation. Despite the well-known clinical benefits of aspirin, a high variability in the antiplatelet effect of aspirin has been reported [6]. This has been associated with a four-fold increased risk of recurrent cardiovascular events [7]. The causes of reduced antiplatelet effect of aspirin are multifactorial and likely comprise clinical, biological, pharmacological, and genetic elements [6,8]. As shown by our group, a reduced antiplatelet effect of aspirin may be related to metabolic disorders including type 2 diabetes (T2D) [9] and an accelerated platelet turnover causing increased numbers of large, immature highly reactive platelets in the circulation [10–12].

In patients with diabetes, hyperglycaemia is associated with increased risk of cardiovascular events [13]. However, the influence of increased levels of haemoglobin A1c (HbA1c) on the antiplatelet effect of aspirin and platelet turnover is unknown in CAD patients with prediabetes. This pertinent issue needs to be addressed as such association may affect the risk of future cardiovascular events.

We hypothesised that elevated levels of HbA1c are associated with increased platelet aggregation and platelet turnover in aspirin-treated CAD patients with T2D and prediabetes. The aim of our work was to investigate HbA1c, platelet aggregation and platelet turnover in a large cohort of stable aspirin-treated CAD patients with and without T2D or prediabetes.

## Methods

In this observational study, we included 865 patients with stable CAD. Among these, 242 (28%) had known T2D and were treated with oral antidiabetic drugs, glucagon-like peptide-1 receptor agonists and/or insulin prior to inclusion. Among CAD patients without diabetes, prediabetes was defined as HbA1c levels between 5.7–6.4% [39–47 mmol/mol] according to the recommendations from the American Diabetes Association [14]. All patients were recruited from the Western Denmark Heart Registry and included at Aarhus University Hospital, Denmark, from November 2007 to January 2011.

Patients were eligible for inclusion if 18 years or older and diagnosed with significant CAD verified by coronary angiography showing at least 50% luminal narrowing in one or more coronary arteries, previous percutaneous coronary intervention or coronary artery bypass grafting. Patients were excluded if they had experienced any ischaemic event or undergone revascularisation within the previous 12 months, had a platelet count <120 x 10^9^/L or >450 x 10^9^/L, or were being treated with anticoagulants or antiplatelet drugs other than aspirin. The present study is a substudy to a large ongoing prospective trial investigating the antiplatelet effect of aspirin in relation to cardiovascular outcomes (Clinical Trials: NCT01383304). Results regarding the HbA1c and prediabetes are novel. Parts of the data (study population, platelet turnover and platelet aggregation) have previously been reported [9,15–18].

All patients were treated with 75 mg non-enteric-coated aspirin daily. Compliance to aspirin treatment was assessed by interview, pill counting, and measurement of serum thromboxane B_2_.

The study was conducted in agreement with the Helsinki Declaration and approved by The Central Denmark Region Committee on Health Research Ethics (project # 2007–0180, 2008–0189, M-2009-0110) and by the Danish Data Protection Agency (# 2011-41-6570). All participants gave written informed consent.

### Blood sampling

Blood samples were obtained with the patients in supine position after 30 minutes of rest. The sample was obtained from an antecubital vein using a large bore needle (19 G), a minimum of venous stasis and vacuum tubes. The blood sample was obtained one hour after intake of the aspirin tablet.

### Assessment of glycaemic state

Glycaemia was evaluated by a single measurement of HbA1c in whole blood using high performance liquid chromatography by Tosoh HLC-723G8 (Medinor A/S, Brøndby, Denmark) and Bio-Rad Variant II (Bio-Rad Laboratories, Copenhagen, Denmark) standardised according to the Diabetes Control and Complications Trial (DCCT) assay. HbA1c was measured in blood drawn at the same time as the platelet function tests were performed.

### Platelet turnover

Platelet parameters were evaluated in whole blood within 90 minutes of blood sampling using an XE-2100 haematology analyser (Sysmex, Kobe, Japan) allowing flow cytometric detection of total and immature platelet counts as previously described [19]. Platelet turnover was evaluated by immature platelet count (IPC), immature platelet fraction (IPF) and mean platelet volume (MPV).

### Platelet aggregation tests

Platelet aggregation was evaluated in whole blood by multiple electrode aggregometry using Multiplate Analyzer (Roche Diagnostics International, Rotkreuz, Switzerland) with arachidonic acid (AA) 1.0 mM and collagen 1.0 μg/mL as agonists and by the VerifyNow Aspirin assay (Accumetrics, CA, USA) as previously described with the modification of using AA 1.0 mM [20,21]. Platelet aggregation was expressed as area under the curve (AUC, aggregation units x minutes [AU*min]) using Multiplate Analyzer and as aspirin reaction units (ARU) using VerifyNow. Blood for platelet aggregation was collected in 3.6 mL (Multiplate Analyzer) and 2.7 mL (VerifyNow) tubes containing 3.2% sodium citrate (Terumo, Leuven, Belgium). Blood samples rested for at least 30 minutes at room temperature but no longer than 2 hours before platelet aggregation analysis.

### Platelet activation and serum thromboxane B2

Soluble serum platelet selectin was determined by ELISA according to the manufacturer’s instructions (R&D systems, MN, USA). Serum thromboxane B_2_ was determined with ELISA (Cayman Chemical, MI, USA) as previously described [11].

### Statistical analyses

All data were tested for normality and equality of variances with appropriate use of log-transformation. Continuous data are presented as mean and standard deviation (SD) if normally distributed, and as median and interquartile range (IQR with 25%; 75% percentiles) if not. Pearson correlation coefficient was used to test for correlation with appropriate use of log-transformation. For continuous variables, a Student´s t-test was used to test differences between two groups. Differences in classifications between two or more groups were evaluated using Fisher´s exact test or the Chi-square test. Two-sided p-values < 0.05 were considered statistically significant. Multiple regression analyses were used to adjust for variables when comparing groups and to identify factors influencing platelet aggregation and platelet turnover.

The primary outcome measure was platelet aggregation induced by AA 1.0 mM. We have previously found that the mean and SD for CAD patients during aspirin therapy is 131 ± 103 AU*min [22]. With a sample size of 865 patients, a significance level (2α) at 5% and a minimal relevant difference at 40 AU*min, we were able to test the hypothesis of increased platelet aggregation in diabetes patients compared with non-diabetes patients with a statistical power of 99%.

## Results

We investigated the antiplatelet effect of aspirin in CAD patients with T2D and prediabetes in comparison to CAD patients without diabetes. Furthermore, we investigated the influence of HbA1c levels on platelet aggregation and turnover.

Clinical characteristics of the study population are shown in Table 1. CAD patients with T2D differed from CAD patients without diabetes with respect to age, body mass index, previous myocardial infarction, bypass surgery, percutaneous coronary intervention, haemoglobin, HbA1c and medical treatment.

**Table 1:** Clinical characteristics of the study population of coronary artery disease patients with and without known type 2 diabetes, n = 865.

	Coronary artery disease without diabetes	Coronary artery diseasewith type 2 diabetes	
*Demographics*	n = 623	n = 242	p-value
Age, years	64 ± 10	66 ± 8	0.001
Female, n (%)	115 (18)	51 (21)	0.38
*Risk factors*			
Current smokers, n (%)	167 (27)	55 (23)	0.23
Body mass index, kg/m2	27 (25; 29)	29 (27; 32)	<0.0001
Hypertension (a)	348 (56)	134 (55)	0.90
*Morbidity*			
Myocardial infarction	567 (91)	166 (69)	<0.001
By-pass surgery	52 (8)	48 (20)	<0.001
Percutaneous coronary intervention	610 (98)	222 (92)	<0.001
Stroke	32 (5)	19 (8)	0.13
*Biochemistry*			
Blood-Haemoglobin, mmol/L	8.9 ± 0.7	8.7 ± 0.7	<0.0001
Estimated GFR, ml/min	80 ± 19	80 ± 77	0.88
Blood-Haemoglobin A1c, %,	5.7 (5.4; 5.9)	7.3 (6.7; 8.1)	<0.0001
Blood-Haemoglobin A1c, mmol/mol	39 (36; 41)	59 (50; 65)	<0.0001
*Medication*, *n (%)*			
Lipid-lowering drugs	571 (92)	218 (90)	0.65
Antihypertensive drugs	554 (89)	229 (95)	0.01
Proton pump inhibitors	61 (10)	35 (15)	<0.05
Insulin	0 (0)	81 (33)	<0.001
Metformin	0 (0)	166 (69)	<0.001
GLP-1 RA	0 (0)	5 (2)	0.80
Any oral antidiabetic drug	0 (0)	116 (48)	<0.001

(a) Hypertension defined as systolic blood pressure ≥ 140 and/or diastolic pressure ≥ 90 mmHg

GFR: Glomerular filtration rate; GLP-1 RA: Glucagon-like peptide-1 receptor agonists

### Platelet aggregation and turnover in CAD patients with T2D

CAD patients with known T2D (n = 242) had increased levels of platelet aggregation compared with non-diabetic CAD patients (n = 623) evaluated by AA- (183 (111; 279) vs 143 (86; 219) AUC, p<0.0001) and collagen-induced platelet aggregation (292 (185; 442) vs 264 (173; 381) AUC, p<0.01) and the VerifyNow Aspirin (446 ± 43 vs 432 ± 35 ARU, p<0.0001) and increased levels of platelet activation evaluated by soluble P-selectin (79 ±26 vs 72 ±25 ng/mL, p<0.001). Platelet count did not differ between diabetic and non-diabetic CAD patients (228 (191; 275) vs. 226 (194; 261) x 10^9^/L, p = 0.26). Platelet turnover was increased in CAD patients with known T2D evaluated by IPC (6.5 (4.8; 8.9) vs. 5.9 (4.4; 7.9) 10^9/L, p<0.01) and by IPF (2.6 (2.0; 4.1) vs. 2.6 (1.9; 3.5) %, p<0.05), but not by MPV (10.9 ±0.9 vs. 10.9 ±0.9 fL, p = 0.52), compared with CAD patients without known diabetes. When adjusting for platelet count alone as well in combination with age and gender, the influence of T2D remained significant for all parameters of platelet aggregation (p-values <0.03), soluble P-selectin (p-values <0.001) and immature platelet count and fraction (p-values ≤0.001). A statistical model including all demographic data that differed between diabetic and non-diabetic CAD patients in Table 1 (age, sex, body mass index, previous myocardial infarction, by-pass surgery, percutaneous coronary intervention, haemoglobin, HbA1c, treatment with antihypertensive drugs, proton pump inhibitors and antidiabetic drugs) showed that the influence of T2D remained significant for collagen-induced platelet aggregation and soluble P-selectin (all p-values <0.05), but not for AA-induced platelet aggregation (p = 0.07), the VerifyNow Aspirin (p = 0.19) and parameters of platelet turnover (p-values > 0.05).

Multiple regression analyses (including age, sex, smoking, diabetes, HbA1c and platelet count) were used to investigate determinants of platelet aggregation and platelet turnover. Diabetes and HbA1c showed significant interaction. Both diabetes and HbA1c levels, together with smoking and platelet count, significantly influenced both AA-induced aggregation and platelet turnover by IPC.

### Platelet aggregation and turnover in CAD patients with prediabetes

Among CAD patients without previously known diabetes and naive to antidiabetic treatment (n = 620, 3 HbA1c values missing) 303 patients (49%) were classified with prediabetes defined as HbA1c levels between 5.7–6.4% [39–47 mmol/mol] [14] and 307 patients (49%) with non-diabetes with HbA1c levels < 5.7% [< 39 mmol/mol]. Ten patients (2%) had new, previously unknown, diabetes defined as HbA1c ≥ 6.5% [≥ 48 mmol/mol], and were excluded from the analyses comparing non-diabetic CAD patients with prediabetic CAD-patients (see below).

Clinical characteristics of the CAD patients without and with prediabetes are shown in Table 2. Patients with prediabetes were three years older, smoked more often, had higher body mass index and reduced kidney function and were more often treated with proton pump inhibitors compared with patients without diabetes.

**Table 2:** Clinical characteristics of coronary artery disease patients with and without prediabetes, n = 620.

	Non-DM n = 307	Prediabetes n = 303	
	HbA1c < 5.7%	HbA1c 5.7–6.4%	
*Demographics*	HbA1c [< 39 mmol/mol]	HbA1c [39–47 mmol/mol]	p-value
Age, years	62 ± 10	65 ± 9	<0.001
Female, n (%)	48 (16)	66 (22)	0.05
*Risk factors*			
Current smokers, n (%)	66 (22)	94 (31)	<0.01
BMI, kg/m2	27 (3)	28 (4)	<0.001
Hypertension (a)	166 (54)	176 (58)	0.32
*Morbidity*			
Myocardial infarction	280 (91)	274 (90)	0.74
By-pass surgery	22 (7)	30 (10)	0.23
Percutanous coronary intervention	300 (98)	297 (98)	0.80
Stroke	15 (5)	15 (5)	0.99
*Biochemistry*			
Blood-Haemoglobin, mmol/L	9.0 ± 0.6	8.9 ± 0.7	0.03
Estimated GFR, mL/min	82 ± 19	78 ± 19	<0.01
Blood-Haemoglobin A1c, %	5.4 (5.4; 5.5)	5.9 (5.8; 6.1)	<0.0001
Blood-Haemoglobin A1c, mmol/mol	36 (35; 37)	41 (40; 43)	<0.0001
*Medication*, *n (%)*			
Lipid lowering drugs	281 (92)	279 (92)	0.81
Antihypertensive drugs	269 (88)	275 (91)	0.21
Proton pump inhibitors	21 (7)	38 (13)	0.02
Antidiabetic drugs	0 (0)	0 (0)	

(a) Hypertension defined as systolic blood pressure ≥ 140 and/or diastolic pressure ≥ 90 mmHg

HbA1c: Haemoglobin A1c; GFR: Glomerular filtration rate

**Fig 1:**
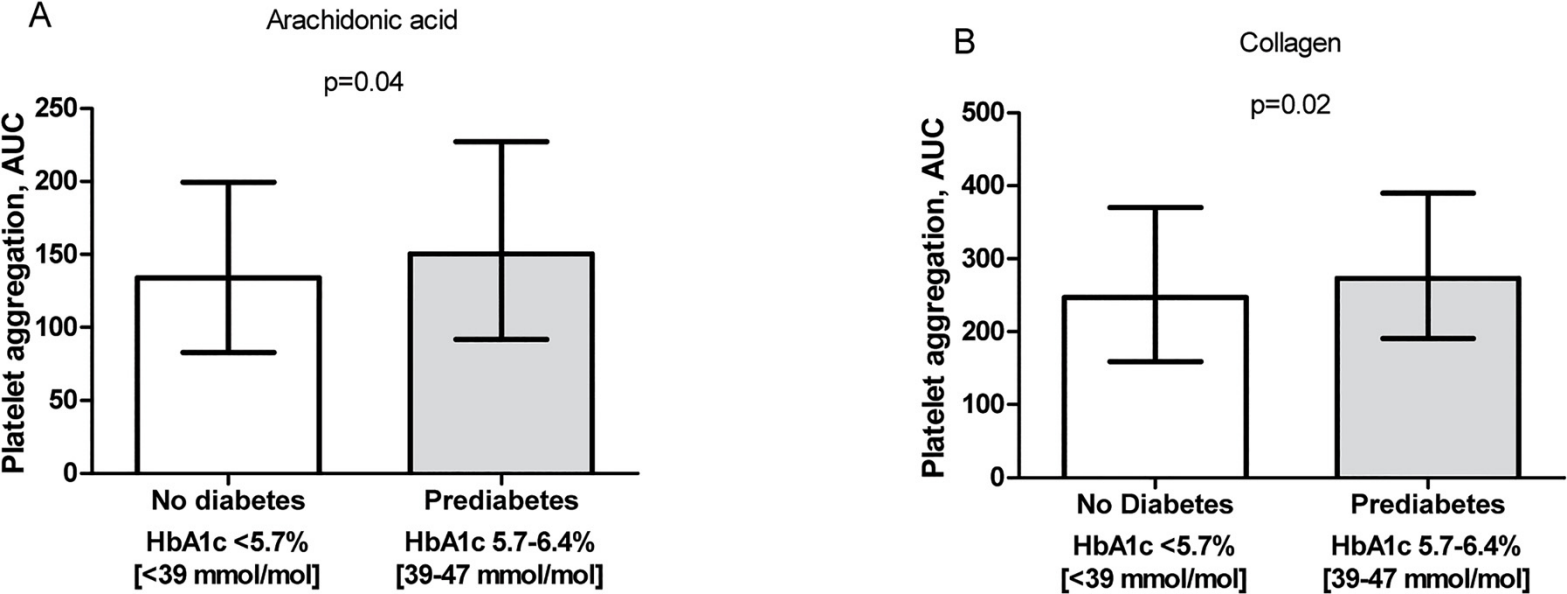
Platelet aggregation. Platelet aggregation in patients with coronary artery disease; 307 patients without diabetes (HbA1c < 5.7%) and 303 patients with prediabetes (HbA1c 5.7–6.4%). Platelet aggregation induced by A) arachidonic acid and B) collagen as agonists using Multiplate Analyzer. Median and interquartile ranges are indicated.

Among patients naive to antidiabetic treatment, CAD patients with prediabetes had significantly increased levels of platelet aggregation evaluated by AA (p = 0.04) and collagen (p = 0.02) as compared with non-diabetic patients (Fig 1). Using the VerifyNow Aspirin assay there was non-significantly higher level of platelet aggregation in prediabetic CAD patients than in non-diabetic patients (434 ± 35 vs. 429 ± 33 ARU, p = 0.12). Platelet count was significantly increased in prediabetic patients (232 (199; 267) vs. 221 (191; 255) x 10^9^/L, p = 0.02), but there was no difference in levels of soluble P-selectin (73 ± 26 vs. 72 ± 24 ng/mL; p = 0.49) compared with the non-diabetic group. Prediabetic patients had numerically higher platelet turnover compared with non-diabetic patients evaluated by IPC (6.0 (4.5; 8.0) vs. 5.7 (4.2; 7.6) x 10^9^/L, p = 0.17), but the values were similar as regards IPF (2.6 (1.9; 3.5) vs. 2.5 (1.9; 3.5) %, p = 0.93) or MPV (10.9 ± 0.9 vs. 10.9 ± 0.8 fL, p = 0.82). When adjusting for platelet count alone as well in combination with age and gender, the influence of prediabetes on platelet aggregation (p-values >0.07), soluble P-selectin (p-values >0.97) and immature platelets (p-values >0.24) became/remained non-significant. Based on differences in demographic data in Table 2 between non-diabetic and prediabetic CAD patients, the influence of prediabetes on platelet aggregation and platelet turnover was investigated in a multivariate regression model including age, sex, smoking, body mass index, previous myocardial infarction, haemoglobin, kidney function, HbA1c, platelet count and treatment with proton pump inhibitors. When adjusting for these variables, prediabetes did not significantly influence platelet aggregation or platelet turnover (p-values > 0.05), while platelet count remained an independent determinant of AA- and collagen-induced platelet aggregation, P-selectin and parameters of platelet turnover including IPC, IPF and MPV (p-values < 0.05).

### Associations between HbA1c levels, platelet aggregation and platelet turnover

**Fig 2:**
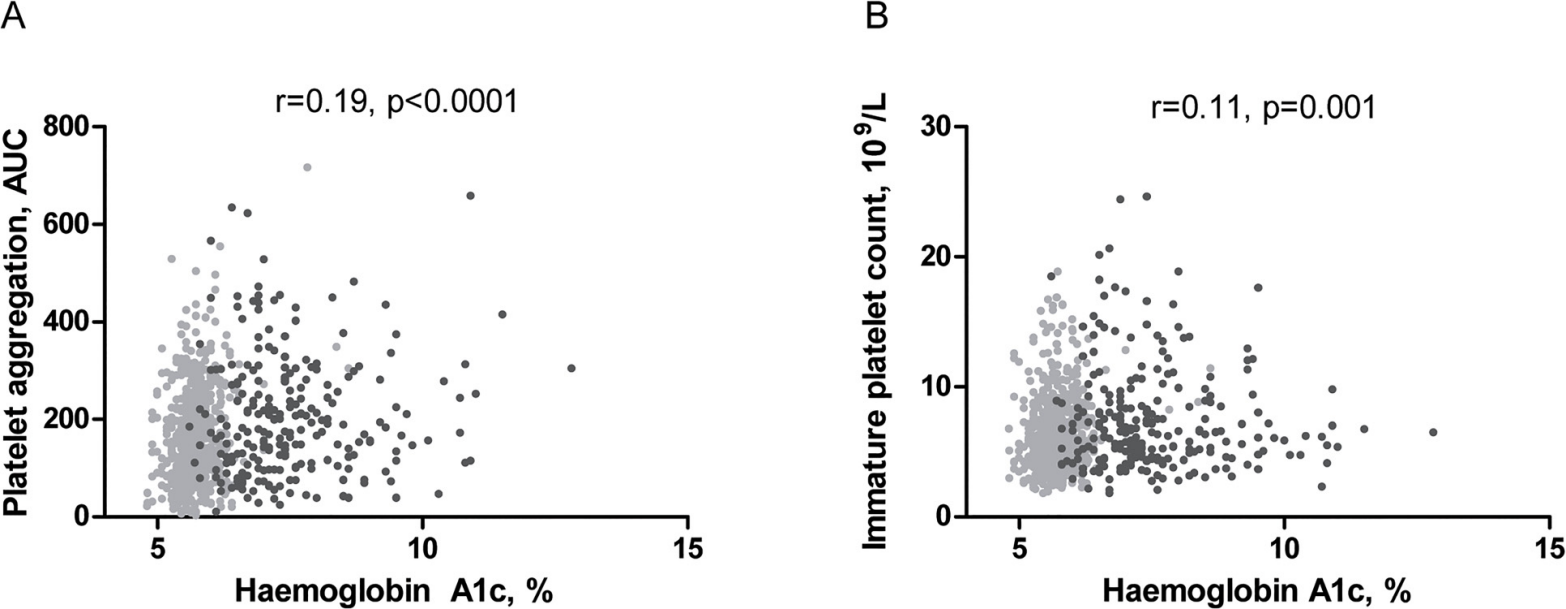
Correlation between haemoglobin A1c and platelet aggregation and platelet turnover. Correlation between HbA1c and A) platelet aggregation induced by arachidonic acid 1.0 mM and B) immature platelet count in patients with coronary artery disease (n = 861). Patients without diabetes (no antidiabetic drugs, n = 620) are marked with light grey and patients with type 2 diabetes (on antidiabetic drugs, n = 241) are marked in dark grey.

In the whole cohort of CAD patients, increased levels of HbA1c correlated significantly, though weakly, with platelet aggregation induced by AA (r = 0.19, p = 0.0001, Fig 2) and collagen (r = 0.10, p<0.01) and the VerifyNow Aspirin (r = 0.15, p<0.0001). Levels of HbA1c correlated positively with soluble P-selectin (r = 0.15, p<0.0001) and platelet count (r = 0.08, p = 0.01). Furthermore, HbA1c levels correlated significantly, though weakly, with increased platelet turnover assessed by IPC (r = 0.11, p = 0.001, Fig 2), but not IPF (r = 0.06, p = 0.08) and MPV (r = 0.04, p = 0.21).

Table 3 shows correlations in CAD patients subdivided into those with and without diagnosed T2D. Interestingly, HbA1c levels only correlated with platelet aggregation, platelet activation, platelet count and platelet turnover in CAD patients without diagnosed diabetes, whereas no correlations were observed in CAD patients with known T2D.

**Table 3:** Correlation between HbA1c and platelet parameters in coronary artery disease patients, n = 861^*^.

	Haemoglobin A1c, %			
	Non-DM; n = 620		T2D; n = 241	
*Platelet aggregation and count*	*r*	*p*-value	*r*	*p*-value
*Multiplate Analyzer*				
Arachidonic Acid, 1.0 mM	0.15	<0.001	0.06	0.40
Collagen, 1.0 μg/mL	0.13	<0.01	- 0.06	0.33
VerifyNow Aspirin	0.08	0.04	-0.02	0.75
Platelet count, 10^9/L	0.13	0.001	0.04	0.50
***Platelet activation***				
sP-selectin, ng/mL	0.11	0.01	0.08	0.25
***Platelet turnover***				
Immature platelet count, 10^9/L	0.12	<0.01	-0.06	0.33
Immature platelet fraction, %	0.05	0.22	-0.08	0.22
Mean platelet volume, fL	0.09	0.03	-0.01	0.94

All data are presented with Pearsons correlation coefficient and p-value.

*4 HbA1c values missing

### Compliance

All patients were compliant to aspirin. This was confirmed by serum thromboxane B_2_ levels 27 ng/mL in all patients, corresponding to a more than 95% inhibition of platelet cyclooxygenase-1 activity [23].

## Discussion

This is the largest study to investigate the influence of HbA1c levels on platelet aggregation and platelet turnover in a cohort of stable CAD patients with T2D or prediabetes. A number of important findings emerge from this study: i) prediabetic CAD patients naïve to antidiabetic treatment had increased platelet aggregation and platelet count compared with non-diabetic patients, ii) increased levels of HbA1c correlated positively, though weakly, with increased platelet aggregation, turnover, count and activation; and iii) these associations were mainly observed in CAD patients naïve to antidiabetic treatment.

To our knowledge, this is the first study to evaluate the antiplatelet effect of aspirin and platelet turnover in CAD patients with prediabetes receiving aspirin mono-therapy. We report that CAD patients with prediabetes had significantly increased platelet aggregation and platelet count during aspirin therapy compared with CAD patients without diabetes. Platelet count has been shown to influence platelet aggregation when investigated with impedance aggregometry [24] and light transmittance aggregometry [25]. When adjusting for platelet count, differences in platelet aggregation disappeared between prediabetic and non-diabetic CAD patients. This strongly suggests that the increased platelet aggregation in patients with prediabetes, was related to increased levels of platelet count.

In the present study, we also extend our previous finding [9] by reporting increased platelet aggregation, platelet turnover and activation in CAD patients with T2D compared with CAD patients. Differences remained after adjusting for platelet count, indicating more reactive platelets in patients with diabetes during aspirin therapy independent of platelet count. Thus, patients with diabetes may have hyper-reactive platelets that respond stronger to minor stimuli and therefore may be consumed faster [26]. This consumption may stimulate an accelerated platelet production and release of immature hyper-reactive platelets by still unknown feedback mechanisms [27]. An accelerated platelet turnover may reduce the antiplatelet effect of aspirin by introducing new immature platelets naive to aspirin into the blood stream [11,28–30].

A major strength of the present study is that all patients were treated with 75 mg aspirin daily as mono-therapy and compliance was confirmed by very low levels of serum thromboxane B_2._ The majority of stable CAD patients receive low dose aspirin as mono-therapy. Despite this, previous studies have mainly been performed in patients on dual antiplatelet therapy [31–34] or in diabetic patients without CAD [35–37], and only a limited number of studies have evaluated the antiplatelet effect of aspirin in CAD patients with diabetes on aspirin mono-therapy [38–40]. Furthermore, reports have mainly focused on associations between levels of glycaemic control and the antiplatelet effect of aspirin in diabetic patients on antidiabetic treatment [31–33,35–37], and not on prediabetic patients naïve to antidiabetic treatment.

In the present study, we report that increased levels of HbA1c correlated with increased platelet aggregation, platelet activation and platelet turnover in patients with CAD. Interestingly, these associations were mainly observed in CAD patients without diagnosed diabetes naive to antidiabetic drugs.

Our finding that CAD patients with prediabetes naïve to antidiabetic therapy had increased platelet aggregation compared with non-diabetic patients may partly be explained by the increased HbA1c levels and increased platelet count observed in these patients. The range of HbA1c defining prediabetes is narrow, and even despite this narrow interval of increased HbA1c, we were able to detect increased platelet aggregation and platelet count in prediabetic patients compared with non-diabetic patients below the HbA1c limit for prediabetes.

The regulation of platelet turnover in patients with CAD is complex [41]. The present study adds important new knowledge about an association between increased levels of HbA1c and levels of immature platelets in non-diabetic CAD patients naive to antidiabetic drugs. Only a limited number of studies have investigated the relationship between hyperglycaemia and platelet turnover, and none of these were performed in patients with CAD [42–45]. Furthermore, three of these studies only investigated MPV as a marker of platelet production and activity [42–44]. A strength of our study was the simultaneous evaluation of MPV, IPC and IPF since these markers have all been associated with platelet turnover and thrombotic events [19,46].

Hyperglycaemia has been shown to enhance platelet activation despite aspirin treatment [47]. In the present study, levels of HbA1c did not correlate with platelet aggregation and turnover in CAD patients with known T2D. Similar lack of correlation between glycaemic control and platelet aggregation has been reported in previous studies [32,33,48]. However, patients in these studies were on dual antiplatelet therapy and are therefore not entirely comparable with our study [32,33,48]. Treatment with antidiabetic drugs may partly explain the lack of correlation between HbA1c and platelet aggregation observed in diabetic CAD patients. In the present study, a large part of the T2D patients were treated with metformin. Metformin has been shown to inhibit platelet aggregation [49]. Also, one third of the diabetic patients in the present study were treated with insulin, which may also inhibit platelet aggregation [32]. Thus, influence of antidiabetic drugs on platelet aggregation may explain, why an association between levels HbA1c and platelet turnover and aggregation was mainly observed in CAD patients with prediabetes not receiving antidiabetic treatment.

Soluble P-selectin reflects increased platelet activation, and we observed a positive correlation between increased levels of HbA1c and soluble P-selectin. In support of this, increasing osmolarity due to hyperglycaemia has been proposed to induce activation of platelet glycoprotein IIb/IIIa and P-selectin expression [50]. Furthermore, an up-regulation of P-selectin and the number of GPIIb/IIIa receptors have been reported in CAD patients with diabetes [51].

Considering the increased cardiovascular risk in CAD patients not only with diabetes, but also in patients with prediabetes, our findings of a reduced antiplatelet effect in patients with prediabetes and T2D may have clinical importance and may call for new antiplatelet therapies and treatment strategies in this patient group [28]. For now, aspirin remains a cornerstone in secondary prevention of cardiovascular events [52]. Possible new treatment options include: i) a higher dose of aspirin [38]; ii) dosing of aspirin twice daily [29,40]; iii) intake of aspirin once daily at bedtime instead of in the morning [53] or iv) switching to or adding another antiplatelet agent such as the P2Y12-inhibitors clopidogrel, prasugrel or ticagrelor [54]. Finally, improving glycaemic control may also be a way of reducing platelet aggregation and outcome [55,56].

Some limitations of the study need to be taken into consideration. Firstly, classification of prediabetes was based on a single measurement of HbA1c in patients without diabetes at the time of inclusion. Secondly, an oral glucose tolerance test in addition to HbA1c could have been informative in order to find undetected prediabetes and diabetes in the non-diabetic CAD patients [57]. However, among patients without diabetes, we only detected 10 patients with new diabetes, and therefore we assume that undetected diabetes in this cohort is limited. Finally, due to the cross-sectional design of the study, a definite causal link between hyperglycaemia, platelet turnover and aggregation could not be explored in detail.

## Conclusion

The present study is the largest so far to evaluate the association between HbA1c levels and platelet aggregation and turnover in a population of stable CAD patients. Our results show that elevated HbA1c levels correlated positively, though weakly, with increased platelet aggregation and platelet turnover in aspirin-treated CAD patients. The antiplatelet effect of aspirin may be attenuated in CAD patients with diabetes and prediabetes compared with non-diabetic patients. This may be related to an increased platelet count in prediabetic patients. Future clinical studies exploring new antiplatelet strategies for the prevention of cardiovascular events in CAD patients with prediabetes and diabetes are warranted.
